# Endogenous endophthalmitis complicating infective endocarditis: a multicentre case-matched control cohort

**DOI:** 10.1093/ehjopen/oeaf136

**Published:** 2025-10-17

**Authors:** Iris Layani, Florent Arregle, Sebastian Santos Patarroyo, Aurore Aziz, Julien Mancini, Julien Ternacle, Peter Laursen Graversen, Emil Foesbol, Nuria Fernández-Hidalgo, Marco Tomasino, Antonia Sambola, Audrey Le Bot, Pierre Tattevin, Christophe Tribouilloy, Claire Lucas, Elisabeth Botelho-Nevers, David Boutoille, Mary Philip, Sandrine Hubert, Neil Tadrist, Natacha Stolowy, Nahema Issa, Frédérique Gouriet, Larry M Baddour, Gilbert Habib

**Affiliations:** Department of Cardiology, La Timone Hospital Marseille, Boulevard Jean Moulin 13005, Marseille, France; Department of Cardiology, La Timone Hospital Marseille, Boulevard Jean Moulin 13005, Marseille, France; Division of Public Health, Infectious Diseases and Occupation Medicine, Department of Medicine, Mayo Clinic, 200 First Street SW | Rochester, MN 55905, United States; Department of Ophthalmology, Hospital Nord and Conception, Aix-Marseille University, Bd jean Moulin 13005 Marseille, France; Aix-Marseille University, APHM, INSERM, IRD, SESSTIM, Hop Timone, Public Health Department, BIOSTIC, Boullevard Jean Moulin 13005 Marseille, France; Unité Médico-Chirurgicale des Valvulopathies Hôpital Cardiologique Haut-Lévêque, CHU de Bordeaux, Avenue Magellan, Pessac 33600, France; Centre de Recherche Cardio-Thoracique de Bordeaux, Univ. Bordeaux, INSERM, U1045, Avenue Magellan, Pessac F-33604, France; Department of Cardiology, Copenhagen University Hospital—Rigshospitalet, Blegdamsvej 9 2100, Copenhagen, Denmark; Department of Cardiology, Copenhagen University Hospital—Rigshospitalet, Blegdamsvej 9 2100, Copenhagen, Denmark; Servei de Malalties Infeccioses, Hospital Universitari Vall D’Hebron, Paseo de la Vall d'Hebron, 119-129 Barcelona, Spain; Department of Cardiology, University Hospital Vall D'Hebron, Paseo de la Vall d'Hebron 119-129, Barcelona, Spain; Department of Cardiology, University Hospital Vall D'Hebron, Paseo de la Vall d'Hebron 119-129, Barcelona, Spain; Infectious Diseases and Intensive Care Unit, Pontchaillou University Hospital, rue Abbé Huet 2 r Henri Le Guilloux, Rennes, France; Infectious Diseases and Intensive Care Unit, Pontchaillou University Hospital, rue Abbé Huet 2 r Henri Le Guilloux, Rennes, France; Department of Cardiology, Amiens University Hospital, 1 rond-point du Professeur Christian Cabrol 80054, Amiens, France; Department of Cardiology, La Timone Hospital Marseille, Boulevard Jean Moulin 13005, Marseille, France; Infectious Diseases Department, University Hospital of Saint-Etienne, 25 bd Pasteur, Saint-Etienne cedex 2 42055, Saint Etienne, France; 13CIRI—Centre International de Recherche en Infectiologie, Team GIMAP, Université Jean Monnet, 10, Rue Tréfilerie – CS 8230142023 Saint-Etienne Cedex 2, Université de Lyon, Inserm, U1111, CNRS, UMR530, Saint-Etienne F42023, France; Maladies Infectieuses et Tropicales—CHU de Nantes 1 place Alexis-Ricordeau 44093, Nantes, Cedex; Department of Cardiology, La Timone Hospital Marseille, Boulevard Jean Moulin 13005, Marseille, France; Department of Cardiology, La Timone Hospital Marseille, Boulevard Jean Moulin 13005, Marseille, France; Department of Ophthalmology, Hospital Nord and Conception, Aix-Marseille University, Bd jean Moulin 13005 Marseille, France; Department of Ophthalmology, Hospital Nord and Conception, Aix-Marseille University, Bd jean Moulin 13005 Marseille, France; Service de Médecine Interne et Maladies Infectieuses, Hôpital Saint-André, CHU de Bordeaux, 1 r Jean Burguet, 33000 Bordeaux, France; Institut Hospitalo-Universitaire Méditerranée Infection, Boulevard Jean Moulin, 13005 Marseille, France; Division of Public Health, Infectious Diseases and Occupation Medicine, Department of Medicine, Mayo Clinic, 200 First Street SW | Rochester, MN 55905, United States; Department of Cardiology, La Timone Hospital Marseille, Boulevard Jean Moulin 13005, Marseille, France

**Keywords:** Infective endocarditis, Ocular complications, Cohort, Endophthalmitis

## Abstract

**Aims:**

Endogenous endophthalmitis (EE) is a rarely reported complication of infective endocarditis (IE). In an international series, we sought to determine the clinical and microbiological profile, treatment, and outcome of patients presenting with IE-related EE.

**Methods and results:**

Cases recorded from 2014 to 2023 in nine centres in Europe and the United States were collected. Results were compared to a matched control group.

**Conclusion:**

Sixty-six patients with EE were reported, mean age of 65.2 ± 14.9 years, 71% (*n* = 47) male. Blood cultures were positive in 97% (64 cases) of patients, with a predominance of streptococci (46%, *n* = 30).

As compared with the control group (*n* = 264), the EE group presented with more frequent diabetes (35% vs. 21%, *P* = 0.02), history of cirrhosis (9% vs. 3%, *P* = 0.04), glomerulonephritis (15% vs. 0.4%, *P* < 0.001), embolism before admission (92% vs. 55%, *P* < 0.001), and Janeway lesions (9% vs. 1%, *P* = 0.002). Streptococcal infection (46% vs. 26%, *P* = 0.001) was more frequent and Enterococcal infection (0% vs. 18%, *P* < 0.001) less frequent in the EE group.

The main ocular symptoms were a decrease in visual acuity (96%), red eye (55%), and ocular pain (55%). Treatment of EE consisted of intravitreal antibiotic injection in 55 (83%) patients and vitrectomy in 17 (26%). Improvement of visual acuity was observed in 36 (55%) patients.

**Conclusion:**

EE is a serious complication of IE with severe residual vision impairment. Patients with IE should be evaluated for ocular complications, since early detection of EE is crucial to prevent delays in management and to preserve visual function.

## Graphical Abstract

**Graphical Abstract:**
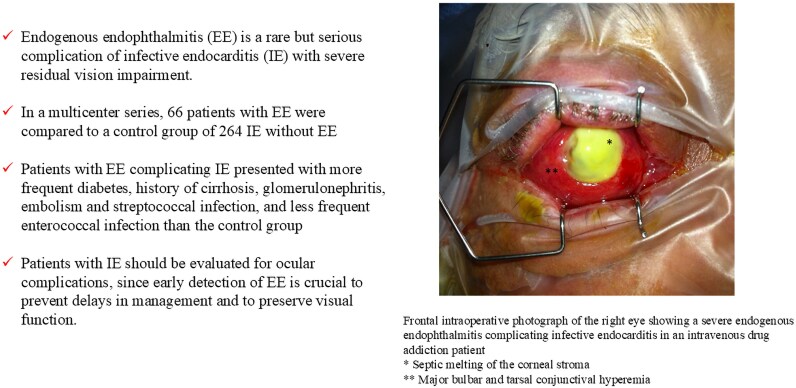

Endogenous endophthalmitis (EE) results from bacterial or fungal infection involving the vitreous and/or aqueous humours.^1^ Although a rare condition, it is frequently complicated by blindness and loss of the affected eye.^2^ Up to 15% of endophthalmitis cases are endogenous, due to haematogenous spread of organisms from extraocular sites, with infective endocarditis (IE) being the most common source.^1,3^ EE resulting from IE has been documented primarily in case reports.^4^ A recent retrospective study focused on predictive factors of EE complicating IE,^5^ but did not include data on microbiology or patient outcomes. In this international investigation, we aimed to determine the clinical features, treatment, and outcomes of patients presenting with EE complicating IE.

## Materials and methods

Cases between 2014 and 2023 were included, and data were collected from the local IE registries of nine hospitals across Europe and the USA (see Supplementary material online, *Table*). Patients were included if they had definite IE according to the Duke^6^ or ESC criteria^7^ and presented with associated EE. Patients were compared to a 4:1 matched control group of IE patients without EE (matching on the nearest date of diagnosis from La Timone, Marseille, IE cohort).

The diagnosis of EE was based on a combination of clinical, microbiological (from blood cultures, intraocular samples from the anterior chamber or vitreous for Gram staining, culture (bacteria or fungi), and/or PCR), and imaging findings. Visual acuity was expressed in logarithms of the Minimum Angle of Resolution (logMAR) units, in accordance with international ophthalmology standards. On this scale, higher logMAR values indicate poorer visual acuity, while lower values (approaching zero or negative) indicate better vision. For reference, 0.0 logMAR corresponds to 20/20 Snellen equivalent, 0.4 logMAR to approximately 20/50, and 1.0 logMAR to approximately 20/200. Severe impairment was defined as visual acuity worse than 0.4 logMAR (≈20/50) in the affected eye. Patients were classified as legally blind when visual acuity was worse than 1.0 logMAR (≈20/200) at follow-up. The thresholds used in this study are consistent with the U.S. definition of legal blindness (best-corrected visual acuity of 20/200 or worse in the better-seeing eye, or visual field ≤ 20 degrees) as established by the Social Security Administration^8^ and are broadly aligned with the World Health Organization (WHO) classification of vision impairment.^9^ For patients with bilateral EE, visual acuity was assessed separately for each eye. For statistical analysis and classification of visual outcomes (e.g. severe impairment, legal blindness), the value from the eye with the worst visual acuity at follow-up was used. This approach was chosen to reflect the maximum degree of functional impairment experienced by the patient and to avoid underestimating vision loss in bilateral cases.

Data collected included demographic information, comorbidities, clinical signs and symptoms, echocardiographic findings, microbiological results, and follow-up information, including ophthalmic surgery and outcome of endophthalmitis (visual recovery, complications, and recurrences).

Categorical variables were expressed as percentages (counts) and compared using Χ² or Fisher’s exact tests. Continuous variables were expressed as mean ± standard deviation (SD) and compared using Student *t*-tests. The visual acuity (logMAR scale) was expressed as median [minimum–maximum]. Analyses were performed using IBM SPSS Statistics 20.0 (IBM Inc., New York, USA).

### Ethical and regulatory aspects

Each centre complied with its national ethical and regulatory standards. All patients included gave consent for this protocol.

## Results

Sixty-six patients with EE complicating IE were included (10 patients from the USA and 56 patients from Europe) and were compared to 264 control patients. Mean age was 65.2 years (± 15), with a male predominance of 71% (*n* = 47). Among comorbidities, EE patients had more diabetes mellitus (35% vs. 21%, *P* = 0.02) and cirrhosis (9% vs. 3%, *P*= 0.04) compared to the control group, but less atrial fibrillation (18% vs. 33%, *P* = 0.02) (*Graphical Abstract*).

### Clinical symptoms of IE

In the EE group, 56 (85%) IE were community-acquired, and 4 patients had injection drug use. More patients in the EE group presented with septic shock (18% vs. 8%, *P* = 0.02). Janeway lesions (9% vs. 1%, *P* = 0.002), glomerulonephritis (15% vs. 0.4%, *P* < 0.001), and systemic embolism (92% vs. 55%, *P* < 0.001) were more frequent in the EE than in the control group (*Table 1*).

**Table 1:** Clinical characteristics, microbiology, echocardiographic findings, and outcome of 66 cases of endophthalmitis complicating infective endocarditis compared to a control group

	Endophthalmitis *n* = 66 (%)	Control *n* = 264 (%)	*P*
Male	47 (71)	195 (74)	0.6
Age^a^	65.2± 14.9	66±14.6	0.8
Medical history			
Hypertension	31 (47)	115 (44)	0.6
Diabetes	23 (35)	55 (21)	**0**.**02**
Smoking	18 (27)	95 (36)	0.16
Atrial fibrillation	12 (18)	88 (33)	**0**.**02**
Chronic renal failure	11 (17)	26 (10)	0.12
Heart failure	7 (11)	49 (19)	0.12
Cirrhosis	6 (9)	8 (3)	**0**.**04**
Intravenous drug use	4 (6)	20 (8)	0.8
Clinical characteristics			
Heart Failure	14 (21)	73 (28)	0.29
Septic shock	12 (18)	22 (8)	**0**.**02**
Osler’s node	4 (6)	5 (2)	0.08
Janeway lesion	6 (9)	3 (1)	**0**.**002**
Glomerulonephritis	10 (15)	1 (0.4)	**<0**.**001**
Total embolism	61 (92)	144 (55)	**<0**.**001**
Echocardiography			
Left heart	59 (89)	223 (85)	0.31
Right heart	5 (8)	47 (18)	**0**.**04**
Prosthetic valve	17 (26)	94 (36)	0.13
Cardiac device-related infective endocarditis	5 (8)	31 (12)	0.33
Vegetation	54 (82)	201 (76)	0.33
Microbiology			
Positive Blood culture	64 (97)	229 (87)	**0**.**02**
*Staphylococcus aureus*	20 (30)	67 (26)	0.43
Streptococci	30 (46)	68 (26)	**0**.**001**
Enterococci	0	48 (18)	**<0**.**001**
Gram negative bacilli	3 (5)	18 (7)	0.78
Fungi	2 (3)	2 (0.8)	0.18

Bold represents significant differences.

^a^Value expressed as mean ± standard deviation.

### Echocardiography

Most patients (*n* = 59, 89%) had left-sided IE, while only 8% had right-sided IE, which was significantly lower than in the control group (18%, *P* = 0.04). There were no cases of bilateral IE. Most patients had native valve IE (68%). Vegetations were identified in 82% of patients, including aortic valve, mitral valve, and tricuspid valve vegetations in 39% (*n* = 22), 49% (*n* = 29), and 9% (*n* = 5), respectively. Annular complications occurred in 17% of patients.

### Microbiology

Blood cultures were positive in 97% of patients in the EE group compared to 87% of patients without EE (*P* = 0.02) and showed a predominance of streptococci and *Staphylococcus aureus*. When compared to the control group, EE patients had significantly more streptococci infection (46% vs. 26%, *P* = 0.001) and less enterococci infection (0% vs. 18%, *P* < 0.001).

### Ocular symptoms, treatment and outcome

EE affected the right eye more frequently (74%, *n* = 49) than the left eye (59%, *n* = 39) and 33% (*n* = 22) of patients had bilateral involvement. EE diagnosis was suspected in the presence of symptoms including vision loss in 96% (*n* = 63) of patients, pain (55%, *n* = 36), and red eye (55%, *n* = 36) (*Table 2* and *Graphical Abstract*).

**Table 2:** Ocular symptoms and outcome

	Endophthalmitis
	*n* = 66 (%)
Ocular symptoms	
Left eye	39 (59)
Right eye	49 (74)
Bilateral	22 (33)
Decreased visual acuity	63 (96)
Ocular pain	36 (55)
Red eye	36 (55)
Conjunctivitis	18 (27)
Hypopion	20 (30)
Tyndall	17 (26)
Hyalitis	20 (30)
Intravitreal haemorrhage	12 (18)
Roth’s spots	16 (24)
Retinal detachment	11 (17)
Visual acuity before treatment (logMAR)^a^	1.5 (0.2–>2.3)
Outcome	
Vision improvement	36 (55)
Visual acuity after treatment (logMAR)^a^	0.7 (0–>2.3)
Severe vision impairment (worse than 0.4 logMar, ≈20/50)	43 (65)
Blindness (worse than 1.0 logMar, ≈20/200)	20 (30)

On the logMAR scale, higher values indicate poorer visual acuity, and values closer to zero (or negative) indicate better visual acuity.

^a^Median [minimum–maximum].

Intravitreal antibiotic injections were performed in 83% (*n* = 55) of patients. Vancomycin was the most frequently used (71%, *n* = 47) antimicrobial, followed by ceftazidime (49%, *n* = 32), and amphotericin B in 6% (*n* = 4) of patients. Vitrectomy was performed in 26% (*n* = 17) of patients.

Vision improvement was observed in 55% (*n* = 36) of patients (74%, *n* = 14 in the *S. aureus* group and 52%, *n* = 15 in the streptococci group). Retinal detachment was present in 11 patients, accounting for 17% of the total cohort. The percentage of patients maintaining a satisfactory level of visual acuity was 30% (*n* = 20). Conversely, 65% (*n* = 43) of patients experienced severe impairment. Among them, 30% (*n* = 20) were classified as legally blind. In-hospital and one-year mortality rates were 16% (*n* = 11) and 27% (*n* = 16), respectively. The IE recurrence rate was 4% (*n* = 2).

## Discussion

This international study provides an analysis of the clinical, microbiological, and outcome characteristics of patients affected by EE in IE. Although this complication is rare, it often leads to significant functional impairment.

General characteristics of patients in our cohort, such as age and sex, were consistent with those of a recent cohort of IE patients.^10^ Comorbidities associated with EE were diabetes mellitus and cirrhosis, as recently reported.^5^

Interestingly, streptococci were the most common pathogens in our cohort, followed by *S. aureus*. No cases of EE complicating *E. faecalis* IE were detected, however. This is an intriguing observation, as most series of IE cases report that enterococci are among the top three IE pathogens.^10^ Enterococci were not only not identified in our current series of EE cases, but were not seen among pathogens in an earlier series of EE cases, where IE was the most common source^3^ or listed as pathogens in a recently published State-of-the-Art review on ocular infections.^11^ Thus, it is tempting to speculate that certain virulence determinants in EE causation may not be present in IE cases due to enterococci and deserve further study.

Unfortunately, at one-year follow-up, about two-thirds of patients had experienced severe residual visual acuity impairment. This highlights the poor functional prognosis of this complication that has been characteristic in other investigations^12^ and warrants evaluation with a thorough history and physical examination in every patient with IE, despite the limited yield in prevalence.^5^

### Limitations

This is a retrospective observational study with inherent limitations, and the small number of patients impairs the robustness of the results. No multivariate analysis was performed due to the small sample size. Moreover, the control group was obtained from a single centre, which could not be representative of epidemiological data from the other centres.

## Conclusion

EE is a rare but serious complication of IE that commonly results in vision loss. Our investigation highlights a high burden of comorbidities such as diabetes mellitus and cirrhosis, and the prevalence of streptococci in this population. Although infrequent, EE should be evaluated in each IE patient with specific evaluation as part of both the history and physical examination, since early detection of EE is crucial to prevent delays in management and to preserve visual function.

## Lead author biography


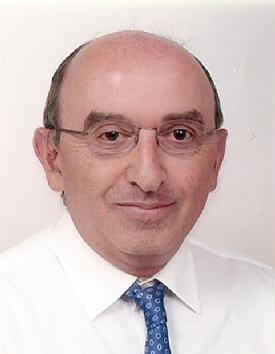


Gilbert HABIB

### Present Position and Address

- Chair of the Cardiology Department (heart failure, valvular heart disease) La Timone Marseille France.
- Director of the echocardiographic laboratory la Timone Marseille.

### European Society of Cardiology Activities

- President of the European Association of Cardiovascular Imaging 2014–2016
- Chairman of the ESC guidelines on infective endocarditis 2009 and 2015
- Chairman of the EACVI guidelines for the use of echocardiography in infective endocarditis 2010
- Chairman of the EORP/ESC EUROENDO registry 2017–2019

### Major Publications

More than 300 publications in international peer-reviewed journals.

## Supplementary Material

oeaf136_Supplementary_Data

## Data Availability

Data, research materials, and analytical methods supporting this study are available through the corresponding author upon reasonable request.
