# Supplementary material for: Endogenous endophthalmitis complicating infective endocarditis: a multicentre case-matched control cohort
Source: Eur Heart J Open. 2025 Oct 17;5(6):oeaf136. doi: 10.1093/ehjopen/oeaf136 (PMC12604093; doi:10.1093/ehjopen/oeaf136)
Supplement: oeaf136_Supplementary_Data [file oeaf136_supplementary_data.docx]

**Supplementary table: Centers included in the study**

| La Timone University hospital, Marseille, France |
| --- |
| Pontchaillou University Hospital, Rennes, France |
| Bordeaux University hospital, Pessac, France |
| Saint-Etienne University hospital, Saint-Etienne, France |
| Nantes University hospital, Nantes, France |
| Amiens University hospital, Amiens, France |
| Vall d’Hebron University hospital, Barcelona, Spain |
| Copenhagen University hospital, Copenhagen, Denmark |
| Mayo Clinic, Minnesota, USA |
